# Ipsilateral Hip Dysplasia in Patients with Sacral Hemiagenesis: A Report of Two Cases

**DOI:** 10.1155/2015/854151

**Published:** 2015-02-09

**Authors:** Tadatsugu Morimoto, Motoki Sonohata, Masaaki Mawatari

**Affiliations:** Department of Orthopaedic Surgery, Faculty of Medicine, Saga University, 5-1-1 Nabeshima, Saga 849-8501, Japan

## Abstract

Sacral agenesis (SA) is a rare condition consisting of the imperfect development of any part of the sacrum. This paper describes two cases of the rare cooccurrence of ipsilateral SA and developmental dysplasia of the hip (DDH) and analyzes possible contributory factors for SA and DDH. Each of a 16-year-old female and 13-year-old female visited our hospital for left hip pain and limping. The findings of physical examinations showed a lower limb length discrepancy (left side) in both cases, as well as left hip pain without limitations of the range of motion or neurological deficits. Initial radiographs demonstrated left subluxation of the left hip with associated acetabular dysplasia and partial left sacral agenesis. MRI revealed a tethering cord with a fatty filum terminale, and periacetabular osteotomy combined with allogeneic bone grafting was performed. After the surgery, the patients experienced no further pain, with no leg length discrepancy and were able to walk without a limp, being neurologically normal with a normal left hip range of motion. The cooccurrence of SA and DDH suggests a plausible hypothesis to explain the embryogenic relationship between malformation of the sacrum and hip.

## 1. Introduction

Sacral agenesis (SA) is a rare condition consisting of the absence, failure of formation, or imperfect development of any part of the sacrum. There are various orthopedic problems associated with SA, including spinopelvic instability, scoliosis, myelomeningocele, a tethering cord, developmental dysplasia of the hip (DDH), knee contractures, and foot deformities [1–5]. However, the etiology of DDH and SA is unknown, and the onset of both diseases may be affected by a number of possible genetic and developmental factors [1–7].

We herein report two cases of the rare cooccurrence of ipsilateral DDH in patients with sacral hemiagenesis who underwent periacetabular osteotomy under spinal anesthesia and analyze possible factors contributing to the development of SA and DDH.

The patients and their family members were informed that data from their cases would be submitted for publication and provided their consent.

## 2. Case 1

**Figure 1:**
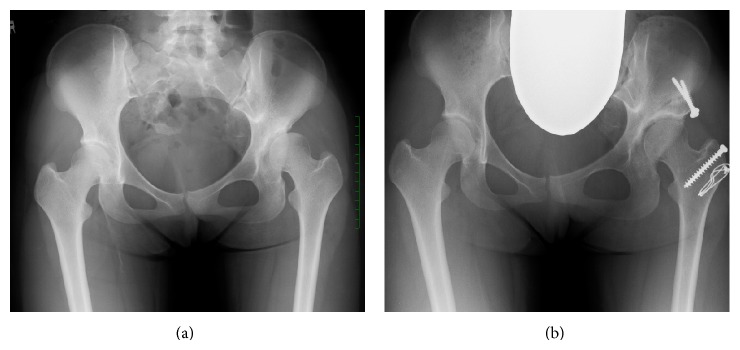
A 16-year-female with left hip subluxation associated with acetabular dysplasia and partial sacral agenesis treated with periacetabular osteotomy combined with allogeneic bone grafting. (a) Preoperative AP radiograph of the pelvis. (b) An AP radiograph obtained two years after the operation. The patient had achieved anatomical correction with an adequate bone stock in the hip joint.

A 16-year-old female presented with a one-year history of left hip pain and limping. She was the product of a normal pregnancy and delivery from a 28-year-old mother who had not been diabetic. The growth and development of the child had been normal, with the exception of treatment with a Pavlik harness due to left developmental dysplasia of the hip beginning three months after birth, followed by open reduction at 14 months of age. The results of a physical examination showed a 1 cm lower limb length discrepancy (left side) and left hip pain, without limitations of the range of motion of the left or neurological deficits. The initial radiographs demonstrated the left hip to be subluxed with associated acetabular dysplasia and partial left sacral agenesis (Figure 1(a)).

**Figure 2:**
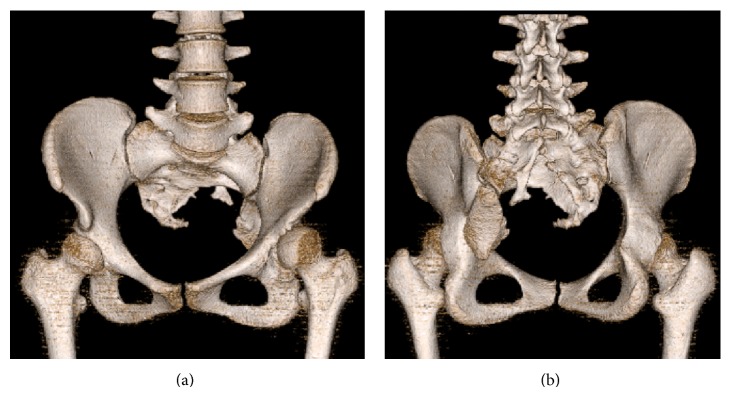
Pelvic three-dimensional CT reconstruction images demonstrated partial left sacral agenesis, in which a rudimentary sacrum had fused with the ilia below the sacroiliac joint. (a) AP view. (b) PA view.

**Figure 3:**
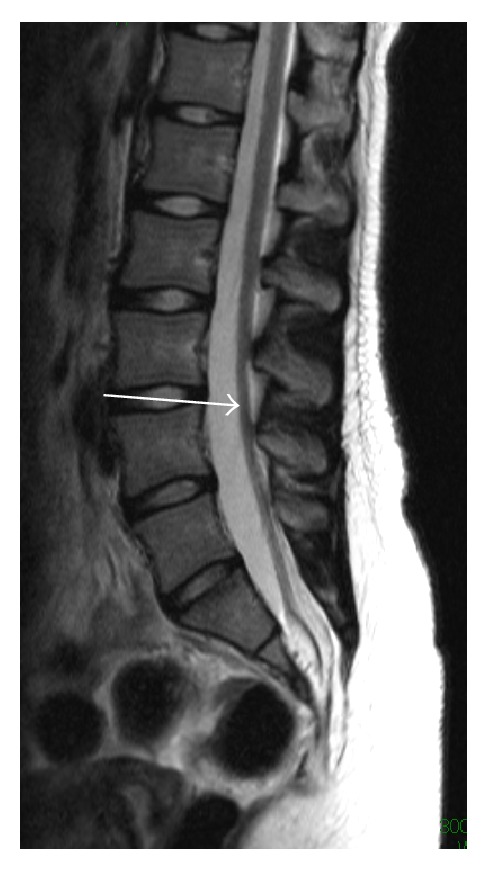
Sagittal T2-weighted magnetic resonance imaging showed a tethering cord (white arrow). The transition between the conus and filum was not obvious.

The dysplasia was concentrically reduced when the extremity was abducted. Pelvic three-dimensional CT reconstruction images clearly demonstrated partial left sacral agenesis, in which a rudimentary sacrum fused with the ilia below the sacroiliac joint (Figure 2) and magnetic resonance imaging (MRI) revealed a tethering cord (Figure 3).

The patient was admitted to our hospital for periacetabular osteotomy which was performed using the insertion of a 27-gauge spinal needle for spinal anesthesia. The anesthesia and surgical procedures were uneventful, and she complained of no neurological deficits after the operation. Two years postoperatively, she underwent the removal of the internal fixation device, as bony fusion was demonstrated radiographically (Figure 1(b)). Currently, at six years postoperatively, she has no pain and is able to walk without a limp, exhibiting neurologically normal findings.

## 3. Case 2

A 13-year-old female visited our hospital for hip pain and limping. She had been born with a low birth weight of 1,400 g via caesarean section due to toxemia in the ninth month of pregnancy. She was the first child born to a 39-year-old mother who was not diabetic. The growth and development of the child had been normal, with the exception of treatment with a Pavlik harness for left developmental dysplasia of the hip beginning three months after birth, which resulted in poor repositioning. At 18 months of age, she underwent open reduction of the hip joint and surgical repair of the sacral dysraphism and lipomeningocele, without any consequent neurological deficits.

**Figure 4:**
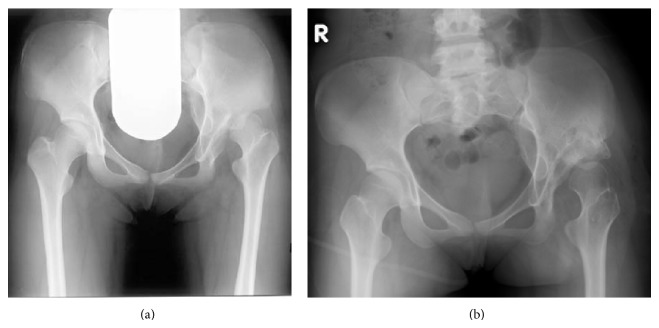
A 13-year-female with left hip subluxation associated with acetabular dysplasia and partial sacral agenesis. (a) A preoperative AP radiograph of the pelvis. (b) An AP radiograph obtained one year after the operation demonstrated partial left sacral agenesis with a rudimentary sacrum had fused with the ilia below the sacroiliac joint.

**Figure 5:**
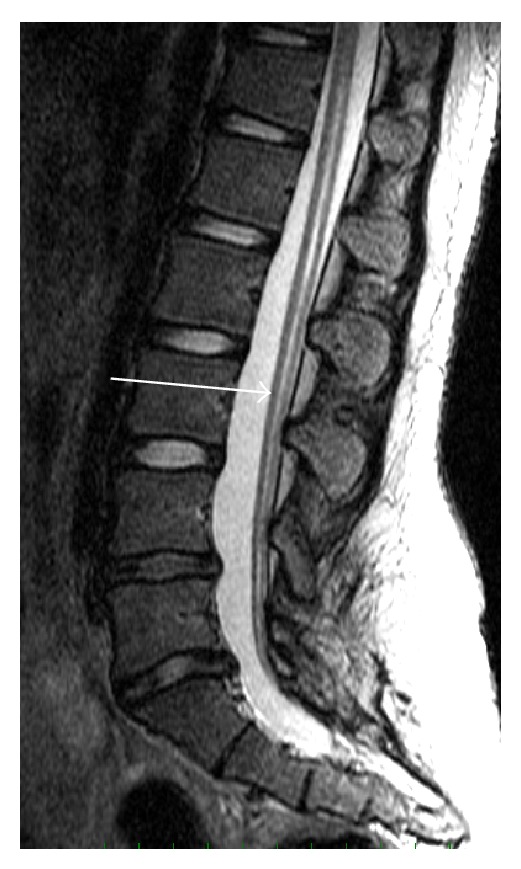
Sagittal T2-weighted magnetic resonance imaging showed a tethering cord with a fatty filum terminale (white arrow).

The results of a pelvic radiograph showed left hip subluxation with associated acetabular dysplasia as well as partial left sacral agenesis (Figure 4(a)). In addition, MRI demonstrated a tethering cord with a fatty filum terminale (Figure 5).

The patient underwent periacetabular osteotomy combined with allogeneic bone grafting with the insertion of a 27-gauge spinal needle for spinal anesthesia. The anesthesia and surgery were uneventful. At that time, a physical examination showed a 3 cm lower limb length discrepancy (left side), painless Trendelenburg gait, and mild limitation of the left hip range of motion, without any neurological deficits.

One year after the surgery, the internal fixation device was removed (Figure 4(b)). Currently, 10 years after the procedure, the patient is able to walk without a limp, with neurologically normal findings and a normal left hip range of motion.

## 4. Discussion

The incidence of DDH in SA patients has been reported to range from 18% to 30% [1, 3], although these reports may not accurately reflect the incidence of mild partial SA, such as that observed in the current two cases. Although the specific etiology of SA remains unknown, the occasional onset of ipsilateral partial sacral agenesis and hip dysplasia, as seen in our cases, suggests that common genetic embryological and/or mechanical factors may be involved in the cooccurrence of SA and DDH.

Embryologically, in the eighth week of gestation, the primary ossification centers in the vertebrae become evident and the hip joint is differentiated from the circumstance musculoskeletal system [7]. The development of the hip joint and fusion of the sacrum may be impaired at that time. Therefore, common genetic factors affecting the gestational development may lead to the development of SA and DDH.

Another theory, that a minor neurological abnormality caused by SA leads to hip dysplasia, may also explain the cooccurrence of SA and hip dysplasia on the ipsilateral side. However, both of our patients had a tethering cord associated with SA and there were no significant positive neurological findings or signs of muscle imbalance preoperatively or during the follow-up period. Furthermore, spasticity or imbalance of the lower extremity muscles due to cerebral palsy may result in progressive subluxation and dislocation of the hip. It has also been reported that more than half of patients with coexisting developmental dysplasia of the hip and spina bifida occulta display had abnormal somatosensory-evoked potentials [8]. Therefore, subclinical muscle imbalances caused by minor neurological abnormalities associated with SA may affect the development of the hip joint [7, 8].

Some investigators have hypothesized that an abnormal glycemic state is a causative factor, based on the association between SA and a high incidence of maternal diabetes [1–5]. However, the true relationship between these conditions is currently unknown, and there was no evidence of maternal diabetes in either of our two cases.

Pang [5] reported that the incidence of dural abnormalities, including tethering and compressive lesions, is 70% among SA patients. Therefore, it is important to check for caudal anomalies on MRI when performing spinal needle insertion for spinal anesthesia. SA patients may be at risk of having a tethering cord, which can result in direct neural damage if the spinal needle is inserted at the lumbar spinal level. For example, Ahmad et al. [9] reported a case of drop foot after spinal anesthesia in a patient with a tethering cord suggestive of needle damage. Fortunately, the spinal anesthesia procedures were uneventful in both of our cases. However, we recommend that general anesthesia without spinal anesthesia should generally be performed in patients with SA due to the potential risk of direct injury to the spinal cord.

It can be difficult to perform a normal periacetabular osteotomy for DDH associated with SA due to the possibility of spinopelvic instability [10]. Richards [10] reported that pelvic osteotomy should be avoided based on the potential of the sacroiliac abnormalities and the resultant rotational instability between the ilium and the sacrum in patients with developmental dysplasia of the hip with partial sacral agenesis and demonstrated excellent results with proximal femoral varus osteotomy in their report. Mesa [11] treated partial SA patients with spinopelvic instability using posterior lumbar iliac percutaneous fusion and pelvic osteotomy with an iliac-bone graft at the osteotomy site. In the current study, the pelvic X-ray and CT findings in both cases showed partial left sacral agenesis, in which a rudimentary sacrum had fused with the ilia below the sacroiliac joint, and it was considered to be a minor possibility of leading to sacroiliac joint instability. Therefore, periacetabular osteotomy was performed, which led to good results in both cases.

In conclusion, the cooccurrence of SA and DDH provides a plausible hypothesis to explain the embryogenic relationship between sacral and hip malformation. Due to the high incidence of dural abnormalities in patients with SA, it is important to perform MRI to check for caudal anomalies and administer general anesthesia (without spinal anesthesia) in SA patients in order to avoid the potential for direct injury to the spinal cord. The clinical and radiographic results of our cases were excellent; however, a further long-term follow-up study on the hip and sacroiliac joints is needed.
